# Alternative Splicing Dynamics Associated with Nutritional Transition and Starvation-Induced PNR in *Leiocassis longirostris* Larvae

**DOI:** 10.3390/biology15131088

**Published:** 2026-07-07

**Authors:** Shanshan Guan, Xiangchao Si, Yongtao Tang, Qiang Li, Chuanjiang Zhou

**Affiliations:** 1 The Observation and Research Field Station of Taihang Mountain Forest Ecosystems of Henan Province, College of Life Sciences, Henan Normal University, Xinxiang 453007, China; 19513039736@163.com (S.G.); sixiangchao@126.com (X.S.); 2 College of Fisheries, Henan Normal University, Xinxiang 453007, China; 3 Fisheries Institute, Sichuan Academy of Agricultural Sciences, Chengdu 611731, China; q_-l@163.com

**Keywords:** *Leiocassis longirostris*, alternative splicing, nutritional transition, starvation, point of no return, differentially spliced events

## Abstract

Alternative splicing (AS) increases transcriptomic diversity; however, its role in teleost larval nutritional physiology remains undetermined. This study investigated alternative splicing (AS) dynamics associated with nutritional transition and the starvation-induced point of no return (PNR) in *Leiocassis longirostris* larvae. Using RNA sequencing and rMATS across eight developmental phases, differentially spliced events (DSEs) and differentially spliced genes (DSGs) were identified between the feeding and starvation trajectories. A total of 84,172 AS events were found, with 93.4% of which were skipped exons (SE). DSEs accumulated in a stage-dependent manner during feeding but increased suddenly under starvation, reaching peak levels at the PNR. DSGs were enriched in cell adhesion, energy sensing, and metabolic reprogramming pathways, with SE splicing most strongly correlated with the progression of starvation. The integration of DSGs with differential exon usage (DEU) revealed 47 PNR-core genes, including *zak*, *lama2*, *mbpa*, and *nhsl2*, which were validated by RT-PCR analysis. The results showed that AS dynamics are associated with stage-dependent regulatory coordination of developmental adaptation and starvation-induced PNR in *L. longirostris* larvae. This study identified molecular targets that may improve larval survival in aquaculture.

## Simple Summary

Fish larvae face a critical survival window when they must switch from relying on their yolk sac to catching and eating food on their own. If they fail to make this transition, they reach a point of no return (PNR) where they can no longer feed and will die. This study investigated how a molecular process called alternative splicing (AS), which allows a single gene to produce different versions of proteins, helps *Leiocassis longirostris* larvae survive this dangerous period. Comparisons between normally fed larvae and starved larvae at various developmental stages revealed that AS patterns significantly changed as starvation advanced, reaching a peak at the PNR. Several key genes involved in stress sensing, tissue structure, and cell survival presented AS patterns under starvation. These findings suggest that monitoring how genes are spliced could help fish farmers identify vulnerable larvae early and develop better feeding strategies to reduce mortality in hatcheries, ultimately supporting sustainable aquaculture production.

## 1. Introduction

In teleost fish, the chances of further development and survival beyond the larval stage are very low. During this period, the transition from endogenous yolk-based nutrition to exogenous feeding plays a key role in survival [1,2]; this transition requires coordinated development of the digestive system, metabolism, and locomotor capacity [3]. In carnivorous species such as *L. longirostris*, this transition is challenging because the yolk is rapidly depleted and the larvae have high energy demands during the early feeding stage.

The economically important long-snouted catfish (*L. longirostris*), also known as Jiangtuan or Feiyu, is a freshwater species endemic to China and is distributed mainly in the Yangtze River, Pearl River, and Heilongjiang River systems [4]. Although this species has been propagated via artificial breeding for decades, larval mortality remains high, mostly because the shift from yolk-dependent nutrition to autonomous feeding has low efficiency, which limits seed production and aquaculture development. The larvae of this species rely on yolk reserves during the first few days after hatching, start mixed feeding around day 4, and exhaust the yolk by day 7. Failure to feed and prolonged starvation can trigger the point of no return (PNR), at which point more than half of the larvae lose the ability to feed and perish. In *L. longirostris*, this threshold occurs on Day 14 post-hatching under standard rearing conditions, as empirically determined through starvation-refeeding experiments in our previous study [5].

Alternative splicing (AS) is a fundamental post-transcriptional mechanism that significantly increases the diversity of transcriptomes and proteomes, allowing one gene to encode multiple protein-coding or regulatory isoforms [6,7,8,9]. This versatility is required for various biological processes in vertebrates, including tissue differentiation, development, and adaptation to environmental stressors [10]. AS plays an important role in teleost embryonic patterning. However, little is known about how AS contributes to this process. RNA sequencing provides a powerful approach to capture transcriptome-wide splicing variation, and rMATS enables the identification of differential splicing events (DSEs) and differentially spliced genes (DSGs) across developmental stages and nutritional conditions with high sensitivity and reproducibility. Therefore, the use of RNA-seq combined with rMATS across eight developmental phases can reveal stage-specific splicing reprogramming associated with successful feeding transition and starvation-induced deterioration.

In this study, high-throughput RNA sequencing and rMATS analysis were conducted to systematically profile the dynamics of AS across eight key developmental milestones in *L. longirostris* larvae, encompassing two distinct physiological trajectories: successful nutritional transitions (F2, F3, F5, F9, and F14), which represent the shift from endogenous yolk dependence to complete exogenous feeding autonomy, and progressive starvation (S5, S9, and S14/PNR), which captures the deterioration cascade from initial mixed nutrition to irreversible PNR. This study was structured around three integrated objectives: (i) comprehensive characterization of the AS repertoire and its temporal evolution during the critical endogenous-to-exogenous nutritional transition; (ii) identification of differentially spliced events (DSEs) and differentially spliced genes (DSGs) that are responsive to nutritional status, thereby distinguishing developmentally regulated splicing from starvation-induced splicing reprogramming; and (iii) identification of the functional networks and biological pathways governed by AS through Gene Ontology (GO) and Kyoto Encyclopedia of Genes and Genomes (KEGG) enrichment analyses, revealing how splicing plasticity supports physiological adaptation.

These findings reveal that AS dynamics are associated with stage-dependent developmental adaptation during feeding establishment and starvation-induced PNR in *L. longirostris* larvae. These results provide novel molecular insights into larval survival strategies during the critical nutritional transition period. As a commercially important carnivorous freshwater species, *L. longirostris* has high aquaculture value, yet larval survival remains limited by poor adaptation to first feeding and starvation sensitivity. Thus, identifying AS-associated regulatory networks may not only advance our understanding of larval developmental biology but also provide candidate molecular targets and biomarkers for future studies on optimizing hatchery management and reducing early-stage mortality.

## 2. Materials and Methods

### 2.1. Experimental Animals

In May 2024, healthy *L. longirostris* larvae were obtained from the Qianwei Branch of Minjiang Port and Shipping Electric Power Development Company (Leshan, China). To ensure optimal physiological performance, water temperatures were maintained within 23.5–26.5 °C. Larvae were sampled at specific days post-hatching, e.g., 2, 3, 5, 9, and 14 dph, representing key developmental stages during the transition from endogenous to exogenous nutrition. Data on larval sizes were obtained in accordance with Reference [5].

### 2.2. RNA Sampling, Extraction, Quantification, and Qualification

**Figure 1:**
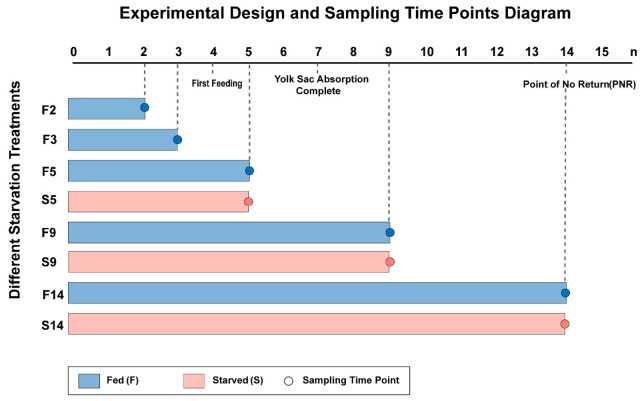
The experimental design and sampling time points are illustrated.

To characterize the changes in the transcriptome of *L. longirostris* in response to nutritional transition and starvation, samples were collected from the feeding (F) and starvation (S) groups at five developmental milestones: Day 2 (F2), Day 3 (F3), Day 5 (F5, S5), Day 9 (F9, S9), and Day 14 (F14, S14) (Figure 1). Day 2 (F2) and Day 3 (F3) corresponded to the yolk-dependent endogenous nutritional phase. Day 5 (F5, S5) corresponds to the mixed nutrition phase (characterized by partial depletion of yolk and initiation of first feeding). Day 9 (F9, S9) corresponds to the complete exogenous nutrition phase (when the yolk is fully depleted). Day 14 (F14, S14) corresponds to the PNR stage for the starvation group, as defined previously by the criterion that more than 50% of the larvae were unable to resume feeding after refeeding. At each sampling time point, 20 larvae were randomly selected from each group, and the larvae sampled at each time point were from the same hatching batch. A total of 160 larvae were used across all groups. After the excess surface moisture was removed from the samples with sterile absorbent paper, they were quickly immersed in RNAlater (Ambion). The samples were equilibrated at 4 °C overnight to ensure complete tissue penetration, after which they were stored at −80 °C. To conduct the transcriptomic analysis, three biological replicates were established at each time point. Each replicate consisted of five larvae (*n* = 15 larvae per group/time point) to minimize biological variation.

Total RNA was extracted from larval samples using TRIzol Reagent (Life Technologies, Carlsbad, CA, USA) according to the manufacturer’s instructions. Briefly, frozen larvae were homogenized in TRIzol reagent, followed by chloroform phase separation and isopropanol precipitation. The RNA pellet was washed with 75% ethanol, air-dried, and dissolved in RNase-free water. The concentration and purity of the RNA were measured using a NanoDrop 2000 (Thermo Fisher Scientific, Wilmington, DE, USA). The integrity of the RNA was evaluated using an RNA Nano 6000 Assay Kit and an Agilent Bioanalyzer 2100 system (Agilent Technologies, Santa Clara, CA, USA). After quality inspection confirmed that the RNA samples met the required standards, RNA-seq library construction and high-throughput sequencing were performed.

### 2.3. Library Preparation for Transcriptome Sequencing

Sequencing libraries were constructed from 1 μg of total RNA per sample using the Hieff NGS Ultima Dual-mode mRNA Library Prep Kit (Yeasen Biotechnology, Shanghai, China). Poly-A mRNA was enriched with oligo (dT) beads, fragmented, and reverse-transcribed into cDNA. After end repair, 3′ adenylation, and adapter ligation, the libraries were size selected using the AMPure XP system (Beckman Coulter, Brea, CA, USA) and amplified via PCR with Phusion High-Fidelity DNA polymerase. Finally, the library quality and insert size were assessed using an Agilent Bioanalyzer 2100 system.

### 2.4. Clustering and Sequencing

Following library quality control (QC), the qualified libraries were pooled based on their effective concentrations and desired sequencing depth. Afterward, the pool was sequenced on an Illumina NovaSeq Xplus platform to generate 150 bp paired-end reads. Raw sequencing data were deposited in the NCBI Sequence Read Archive (SRA) under the BioProject accession number PRJNA1445138.

### 2.5. Quality Control and Sequencing Data Analysis

Raw data in fastq format were initially processed using fastp software (version 0.23.1) [11,12]. Clean data were obtained by removing adapter-containing, poly-N, and low-quality reads from the raw data. Quality metrics, including Q20, Q30, and GC content, were assessed, and only the high-quality clean reads were used for downstream analyses.

Reference genome sequences and corresponding gene model annotation files were downloaded from the genome database. The reference genome version information was from *L. longirostris*. Customer-V1.genome.fa [13]. Clean reads were accurately aligned with the reference genome using HISAT2 (version 2.0.4) software [14], and read mapping information on the reference genome was obtained. Next, the aligned reads were assembled using StringTie (version v2.2.1), and the transcriptome was reconstructed for subsequent analysis [15]. Transcript abundance was quantified using the maximum-flow algorithm in StringTie and normalized as fragments per kilobase of transcript per million mapped fragments (FPKM) [16]. FPKM values were used only for visualization of transcript abundance. This normalization strategy was selected because it accounts for the sequencing depth and gene length, providing a standardized measure that minimizes technical bias during the transition from the endogenous to exogenous feeding stages.

### 2.6. Splicing Event Quantification

**Figure 2:**
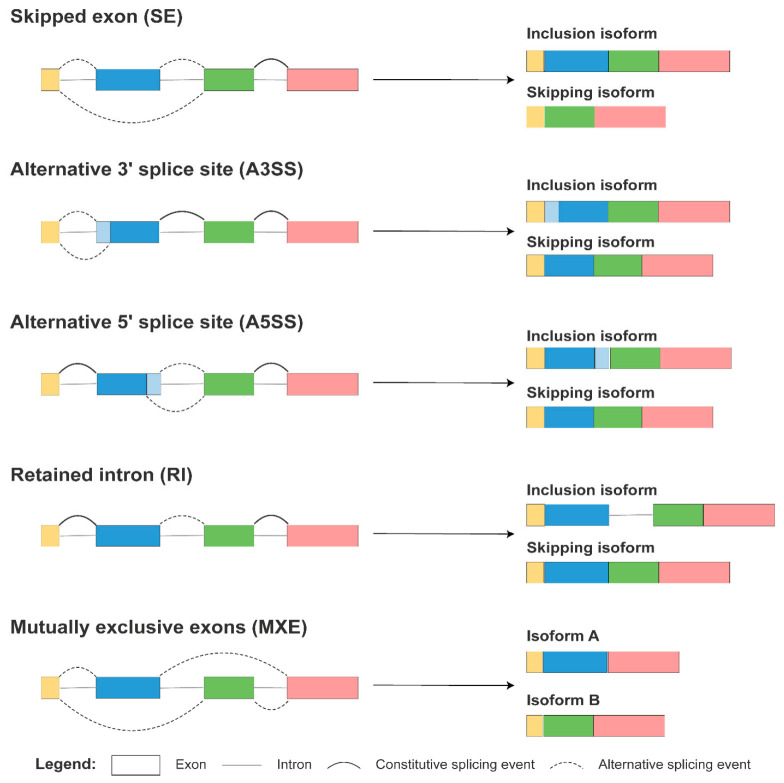
Schematic representation of the five types of AS events: SE, A3SS, A5SS, RI, and MXE. The DNA sequences and the resulting mRNA sequences are shown on the left and right sides of the arrows, respectively. rMATs uses read pair mapping to exon boundaries to identify AS events. Any reads mapped to the exon–exon boundaries involved in a specific splicing event, or those that fell in the alternative exon/intron, were then classified as belonging to the “inclusion isoform” or the “skipping isoform”. The figure was modified from reference [6].

Alternative splicing (AS) events were detected and quantified using rMATS (4.0.2) software [17,18]. rMATS was run using the BAM files from all biological replicates in each condition, allowing estimation of replicate variation and identification of differential splicing events. Two transcript isoforms were quantified for splicing events: the exon inclusion isoform and the exon skipping isoform. The statistical model implemented in rMATS defines the count of reads uniquely mapped to these two isoforms as the inclusion level (IncLevel) of the splicing site. Likelihood-ratio tests were conducted to calculate *p*-values representing intergroup differences in IncLevel, and the Benjamini–Hochberg procedure was applied to adjust raw *p*-values for multiple testing to generate false discovery rate (FDR) values. For the present analysis, the default filtering threshold built into rMATS was initially adopted: an absolute difference in percent spliced-in (PSI) (|ΔPSI|) greater than 0.0001. These genes were classified into five categories based on their splicing pattern: skipped exon (SE), retained intron (RI), alternative 5′ splice site (A5SS), alternative 3′ splice site (A3SS), and mutually exclusive exons (MXE) (Figure 2). Statistical comparisons were performed within three categories as follows: (i) longitudinal developmental comparison, (ii) starvation stress progression, and (iii) cross-stress comparison. The longitudinal developmental comparisons included F3 vs. F2, F5 vs. F3, F9 vs. F5, and F14 vs. F9. The progression of starvation stress was analyzed by comparisons of S5 vs. F3, S9 vs. S5, and S14 vs. S9. Finally, S5 vs. F5, S9 vs. F9, and S14 vs. F14 cross-stress comparisons were conducted.

To screen for significant DSEs, the following stringent criteria were applied: a total RNA-seq read count per sample of at least 10 across all samples within a comparison pair; the filtration of events with an average PSI value below 0.05 or above 0.95 in either of the compared sample groups to account for low or near-fixed splicing levels; and an FDR threshold of ≤0.01; and an absolute difference in PSI (|ΔPSI|) between the comparison groups of at least 0.05. Following deduplication, DSGs were screened.

### 2.7. Differential Exon Usage Analysis

Differential exon usage (DEU) analysis was performed using DEXSeq (version 1.12.2) with default parameters. An adjusted FDR threshold of <0.05 was applied to identify significantly differentially expressed exons between groups. Through this analysis, the differences in expression were investigated at the exon level across samples, thereby providing insights into which exons may contribute to the regulation of functional gene expression. In addition to AS, DEU analysis facilitated a comprehensive understanding of the molecular mechanisms underlying the regulation of gene expression.

### 2.8. GO and KEGG Enrichment Analysis of DSGs

Gene Ontology (GO) functional annotation and enrichment analysis were performed on the DSGs. GO annotation was conducted using InterProScan (version 5.34–73.0) against the GO database (http://www.geneontology.org/). The DSGs were classified into three major GO domains, namely, biological process (BP), cellular component (CC), and molecular function (MF). GO terms with q values < 0.05 were considered to be significantly enriched. Simultaneously, KEGG functional classification was performed using the KEGG database (http://www.genome.jp/kegg/). This analysis was conducted to systematically characterize the functional distribution of DSGs and identify the physiological processes and molecular pathways affected by differential AS events across the comparison groups.

### 2.9. Validation of AS Events

Four randomly selected DSEs were used for validation. Gene-specific primers corresponding to the AS sites were designed using Primer 5.0. The presence of different transcript isoforms was confirmed by RT-PCR followed by agarose gel electrophoresis, with the actin gene serving as the internal control. The sequences of primers used are listed in Table 1.

## 3. Results

### 3.1. Sequencing Data Statistics

After quality feeding, 188.30 Gb of clean sequencing data were generated, and the Q30 base percentage of all the samples was found to be ≥96.87%, and the GC content was consistent across all the samples, which indicated that the sequencing results were of high quality (Table A1) [19]. Table A1 shows high Q30 scores and consistent GC content across all the samples.

### 3.2. Statistical Analysis of Alignment Rates Between Sequencing Data and Reference Genomes

The sequencing data were highly reliable, with mapping efficiencies to the *L. longirostris* reference genome ranging from 90.49 to 95.23%. These rates exceeded the standard threshold for transcriptomic profiling, providing a robust foundation for subsequent bioinformatic analyses [20].

### 3.3. Quality Assessment of RNA-Seq Data Based on FPKM Distribution and PCA

**Figure 3:**
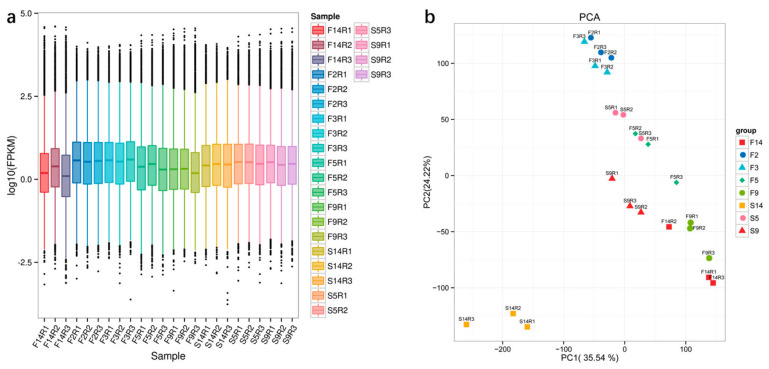
Quality Assessment of RNA-seq Data based on FPKM Distribution and PCA. (**a**) Boxplots show the distribution of log10-transformed FPKM values across all samples. (**b**) Principal component analysis (PCA) revealed clear clustering of samples according to developmental stage and nutritional condition.

To assess the quality and reproducibility of the RNA-seq data, the distribution of FPKM values was examined across all samples. The boxplots showed comparable expression distributions among biological replicates, indicating good data consistency. Principal component analysis (PCA) further revealed clear clustering of samples by developmental stage and nutritional condition, with replicates grouped closely together. These results confirmed the reliability of the transcriptome dataset for subsequent analyses (Figure 3).

### 3.4. Identification, Classification, and Dynamic Changes of DSEs

**Figure 4:**
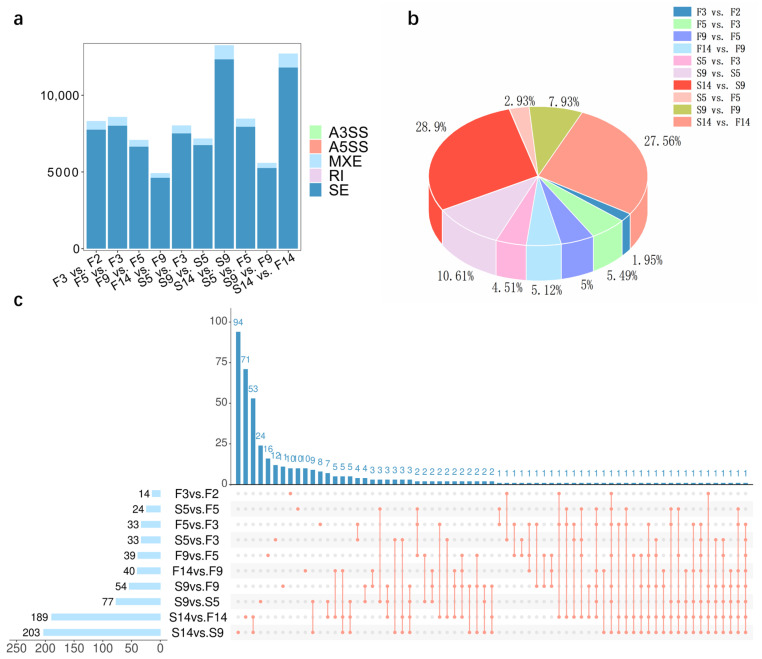
Identification and dynamic changes in DSEs. (**a**) The number of SE, RI, MXE, A5SS, and A3SS events identified in each comparison is illustrated. (**b**) The pie chart shows the proportional distribution of total DSEs across all comparisons. (**c**) The UpSet plot illustrates the intersections of DSGs among different comparisons. The horizontal bar chart (**bottom left**) indicates the total number of DSGs in each comparison, whereas the vertical bar chart (**top**) shows the number of DSGs shared among the comparisons denoted by the connected dots below.

The number of AS events identified was 84,172, among which SE events were the most abundant, accounting for 78,639 events (93.4%). The number of MXE events detected was 5533 (6.6%). However, RI, A3SS, and A5SS events were not detected, reflecting the predominant splicing modes in this species. The distribution of AS event types and the number of AS events identified in each comparison are presented in Figure 4a. SE was the predominant type of AS event. This predominance of SE events is consistent with findings across various species and under various physiological conditions. For example, comprehensive RNA-seq analyses in *Schizosaccharomyces pombe* have revealed widespread exon skipping under various physiological conditions and in transcription-processing and RNA-processing mutants [21]; these findings highlight the broad functional significance of SE in the regulation of gene expression. Similarly, systematic transcriptome profiling of *Chlamydomonas reinhardtii* under nitrogen starvation revealed that SE is the major type of AS that plays key roles in abiotic stress responses [22], indicating that plants also perform SE-mediated regulation to modulate physiological processes during nutrient deprivation.

The criteria for identifying significant DSEs were as follows: an average RNA-seq read count of at least 10 across the two sample groups, exclusion of events with an average PSI value below 0.05 or above 0.95 in either group, an FDR of no more than 0.01, and an absolute value of |ΔPSI| of at least 0.05. After redundant DSEs were removed, the remaining DSEs were collapsed into DSGs, facilitating gene-level analysis of AS. The number of DSEs and DSGs detected in each comparison is summarized in Table 2.

In the feeding group, the number of DSEs increased gradually across developmental transitions, with 16 events identified in F3 vs. F2, 45 events in F5 vs. F3, 41 events in F9 vs. F5, and 42 events in F14 vs. F9. In the starvation group, 37 DSEs were detected in S5 vs. F3, 87 DSEs in S9 vs. S5, and 237 DSEs in S14 vs. S9, indicating a marked increase in splicing alterations at later stages of starvation. When fed and starved larvae were compared across the same developmental stages, 24 DSEs were identified in S5 vs. F5, 65 DSEs in S9 vs. F9, and 226 DSEs in S14 vs. F14. The proportional distribution of total DSEs across all comparisons is shown in Figure 4b. An UpSet plot (Figure 4c) revealed the intersections of DSGs among different comparisons, highlighting shared and unique splicing events across developmental stages and nutritional conditions. The greatest number of DSEs was observed in S14 vs. F9 and S14 vs. F14, suggesting that prolonged starvation induced extensive remodeling of AS patterns. Overall, these results indicated that AS dynamics were stage dependent and strongly affected by starvation, particularly at later developmental stages.

### 3.5. Functional Enrichment Analysis of DSGs During Nutritional Transition

**Figure A1:**
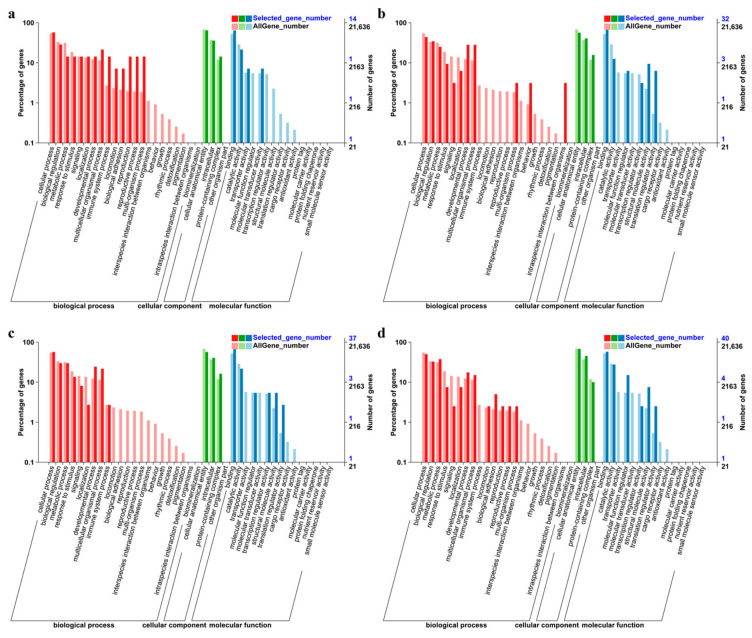
GO functional enrichment analysis of DSGs during nutritional transition. Bar plots (**a**–**d**) illustrate the GO enrichment results for the pairwise comparisons: F3 vs. F3 vs. F2, F5 vs. F3, F9 vs. F5, and F14 vs. F9.

**Figure A2:**
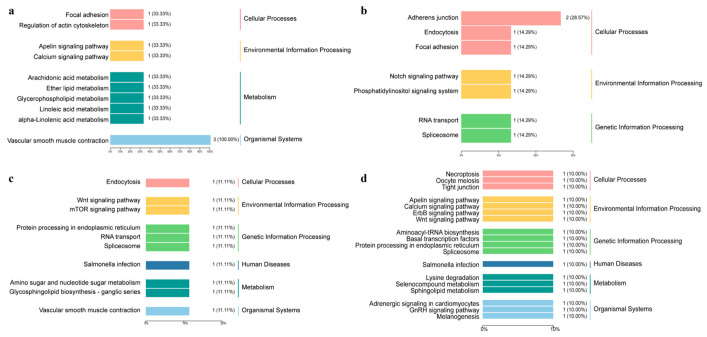
Enriched KEGG pathways of DSGs in nutritional transition. Bar plots (**a**–**d**) show the enriched KEGG pathways of DSGs in the F3 vs. F2, F5 vs. F3, F9 vs. F5, and F14 vs. F9 comparisons.

To investigate the functions of DSGs during nutritional transition, GO (Figure A1) and KEGG (Figure A2) enrichment analyses were conducted for each developmental comparison. Across all four transitions, DSGs were consistently enriched in pathways related to cytoskeletal organization, cell adhesion, and intracellular signaling (e.g., focal adhesion, actin cytoskeleton regulation, calcium signaling, and phosphatidylinositol signaling). This similarity in pathway enrichment reflects the necessary structural and metabolic reprogramming as the teleost digestive tract evolves from a simple, undifferentiated tube to a segmented, functional apparatus. These shared pathways suggest that AS plays a fundamental regulatory role, orchestrating the morphological remodeling and cellular communication required for larvae to adapt to physiological shifts.

During the yolk-dependent phase, enrichment in lipid metabolism (e.g., arachidonic acid, linoleic acid, and glycerophospholipid metabolism) and cardiovascular/cytoskeletal pathways reflects the substantial mobilization of yolk-oil reserves [23]. The presence of apelin signaling before the onset of first feeding indicates maturation of the cardiovascular system to meet increasing metabolic demands. Apelin, a bioactive peptide, and its receptor (APJ) play key roles in cardiac function and vascular development [24]. Early ontogeny prioritizes the establishment of locomotor capacity, which is important for the future pursuit of prey and the prevention of predators under current conditions [25]. AS-mediated regulation of muscle contraction and calcium signaling supports the preparatory maturation of the buccopharyngeal and locomotor structures required before the mouth opened on Day 4.

As larvae start feeding, the focus shifts to adherens junctions, Notch signaling, endocytosis, the spliceosome, and RNA transport. This stage represents a survival bottleneck in which larvae transition from depending on the yolk to actively capturing prey. The enrichment of adherens junctions reflects the establishment of epithelial barriers for gut integrity [26], with *Llo_GLEAN_10013885*, which encodes an LMO7-like protein that functions in cytoskeletal organization and cadherin–actin linkage, and *Llo_GLEAN_10015332*, which encodes the tyrosine-protein kinase Fyn and probably mediates phosphorylation-dependent regulation of adherens junction assembly, thereby contributing to cytoskeletal organization and junctional signaling, respectively. Endocytosis is consistent with dominant pinocytotic digestion before the development of gastric glands. Concurrently, Notch signaling drives the differentiation of the intestinal epithelium, coordinating the rapid segmentation of the digestive tract necessary for processing the first ingested exogenous food [27].

By Day 9, the yolk is completely depleted, and the larvae rely entirely on external food. Enrichment in mammalian target of rapamycin (mTOR) and Wnt signaling, along with membrane biosynthesis (e.g., glycosphingolipid and amino sugar metabolism), indicates a shift from morphogenesis to nutrient-dependent growth control and the fine-tuning of digestive surfaces [28,29]. Enrichment of infection/immune-related gene sets indicates that stress-associated immune activation occurs following the developmental maturation of the intestinal epithelial structure, which is required for the digestion of exogenous food. The enriched gene *Llo_GLEAN_10000985* has multiple functional annotations involving cytoskeleton and signal transduction related to stress fiber assembly and infection.

The most extensive functional diversification emerged in the stabilization phase. Enrichment in protein synthesis (aminoacyl-tRNA biosynthesis) and barrier maturation (tight junction) reflects the attainment of long-term physiological homeostasis. The maturation of tight junctions is important for establishing a strong intestinal barrier, maintaining normal digestion, and facilitating the orderly transport and excretion of waste. Apelin signaling occurs again, likely reflecting the continued coordination between the cardiovascular system and metabolic processes to support sustained growth and the transition toward the juvenile stage. This stage-dependent remodeling in *L. longirostris* is similar to that observed in other teleosts, such as *Gadus morhua L*. [30], in which digestive maturation is completed during the transition to autonomous feeding. The sequential enrichment pattern in *L. longirostris* revealed a coherent survival strategy, progressing from energy mobilization and locomotor preparation to the morphogenesis of the digestive tract, metabolic tuning, immune surveillance, and finally to the stabilization of the barrier and sustained growth.

### 3.6. GO and KEGG Enrichment Analysis of Differentially Spliced Genes in the Starvation Group

**Figure A3:**
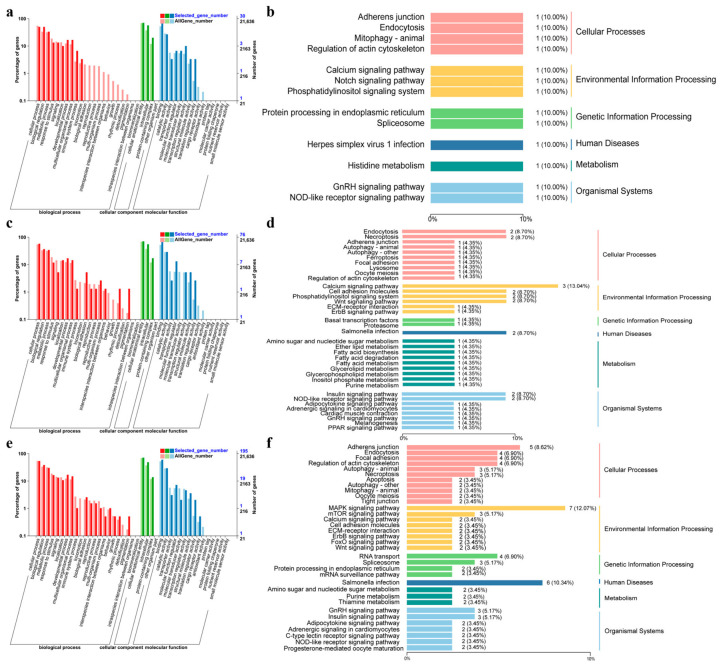
GO and KEGG enrichment analyses of DSGs in the starvation group. (**a**,**c**,**e**) The bar plots illustrate the GO enrichment of DSGs in the S5 vs. F3, S9 vs. S5, and S14 vs. S9 comparisons, respectively. (**b**,**d**,**f**) The enriched KEGG pathways of DSGs in the S5 vs. F3, S9 vs. S5, and S14 vs. S9 comparisons, respectively, are illustrated.

To investigate the biological functions of DSGs under starvation conditions, GO and KEGG enrichment analyses were performed for the starvation group across three developmental comparisons (S5 vs. F3, S9 vs. S5, and S14 vs. S9) (Figure A3).

Throughout the starvation period, pathways governing cell adhesion and tissue remodeling (e.g., adherens junction, cell adhesion molecules, focal adhesion, and ECM-receptor interaction) were consistently enriched, indicating the progressive disassembly of the gut epithelium with the advancement of nutrient deprivation. Simultaneously, the recurring presence of mitochondrial/autophagic clearance (mitophagy, autophagy, and phagosome) and energy-sensing signaling (insulin, mTOR, and phosphatidylinositol signaling) indicated sustained metabolic reprogramming. These conserved themes suggest that AS acts as a coordinated regulator, orchestrating the gradual erosion of tissue.

During the early starvation phase (S5 vs. F3), the DSGs were enriched in genes related to histidine metabolism and mitophagy, indicating a rapid shift from growth-related processes toward energy conservation. In milkfish, histidine is the most abundant free amino acid in white muscle and decreases considerably during prolonged starvation [31]; this shift suggests that it may be mobilized to support energy metabolism under food deprivation. In the mid-starvation phase (S9 vs. S5), pathways such as fatty acid degradation, the proteasome, and insulin signaling became highly active, reflecting a transition to bulk lipid and protein catabolism. At this stage, larvae mobilize muscle and liver reserves, while altered insulin signaling promotes metabolic adaptation, and necroptosis eliminates damaged cells to limit systemic inflammation. In the PNR stage (S14 vs. S9), DSGs were more strongly enriched in adherens junction, MAPK signaling, endocytosis, Wnt signaling, and mTOR signaling, suggesting severe disruption of cell adhesion, stress signaling, and nutrient sensing. Enrichment of infection/immune-related gene sets was enriched in both S9 vs. S5 and S14 vs. S9, with a greater number of associated genes in the latter comparison, indicating a progressive intensification of immune-related processes during the late starvation phase. This pattern suggests that prolonged nutrient deprivation may initially suppress baseline immune function, thereby triggering compensatory activation of innate defense pathways as a homeostatic protective response. The detection of this pathway in the fed F9 control indicates that it does not occur exclusively under starvation but is rather a shared response module present during normal development that becomes more pronounced under starvation [32]. These results indicate that starvation elicits a staged and conserved adaptive response in this species, beginning with the mobilization of amino acids and followed by enhanced breakdown of lipids/proteins and activation of stress-related and immune-related pathways. During prolonged starvation, *solute carrier family 25 member 3b isoform X2* (*slc25a3b*), *Llo_GLEAN_10012656*, *myosin binding protein Hb isoform X1* (*mybphb*), and *Llo_GLEAN_10023520* remained persistently responsive, suggesting that they may play important roles in starvation tolerance. SLC25A3b is primarily associated with mitochondrial phosphate transport and energy production, *Llo_GLEAN_10012656* is associated with cell cycle regulation and programmed cell death, *mybphb* is associated with the structural maintenance and remodeling of muscles, and *Llo_GLEAN_10023520* may participate in translational regulation and the suppression of protein synthesis. These persistently responsive genes are involved in the coordinated regulation of energy metabolism, cellular homeostasis, muscle remodeling, and anabolic inhibition during long-term starvation.

### 3.7. Comparative Functional Enrichment Analysis of DSGs Between Starvation and Feeding Groups at Synchronous Developmental Stages

**Figure A4:**
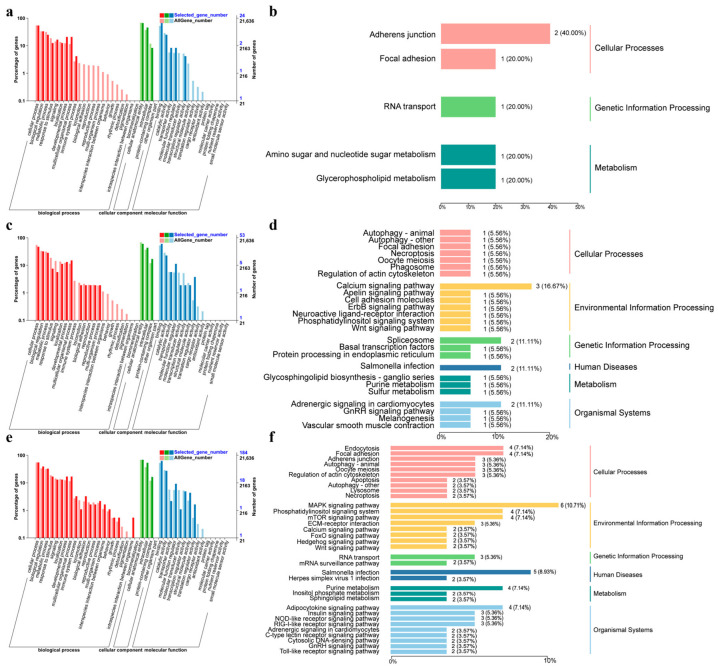
Comparative functional enrichment analysis of DSGs between the starvation and feeding groups at synchronous developmental stages. (**a**,**c**,**e**) The bar plots illustrate the GO enrichment of DSGs in the S5 vs. F5, S9 vs. F9, and S14 vs. F14 comparisons, respectively. (**b**,**d**,**f**) The enriched KEGG pathways of DSGs in the S5 vs. F5, S9 vs. F9, and S14 vs. F14 comparisons, respectively, are illustrated.

To investigate the functional consequences of AS under starvation, GO and KEGG enrichment analyses were performed on DSGs between the starvation (S) and feeding (F) groups at synchronous developmental stages (S5 vs. F5, S9 vs. F9, and S14 vs. F14). The results revealed stage-specific functional remodeling of DSGs in response to starvation (Figure A4).

Comparative analysis of DSGs between starved and fed larvae at synchronized developmental stages revealed that starvation induces stage-specific functional remodeling. At the early stage, DSGs were enriched mainly in the terms “adherens junctions”, “focal adhesion”, “RNA transport”, and “glycometabolic”, indicating initial disturbance of membrane organization and cell–cell communication. In the middle stage, autophagy, necroptosis, the spliceosome, ER protein processing, and multiple signaling pathways became prominent, suggesting a transition toward stress adaptation, transcriptomic reprogramming, and protein quality control. At the late stage, enrichment expanded to metabolic, proteolytic, and nutrient-sensing pathways, including amino acid, lipid, and carbohydrate metabolism, proteasome, endocytosis, and major signaling pathways, such as the MAPK signaling pathway, Wnt signaling pathway, phosphatidylinositol signaling system, and mTOR signaling pathway, reflecting a broad starvation-induced shift toward survival maintenance. In addition to the results from normal developmental and starvation-only comparisons, these findings indicate that AS participates in both maturation and adaptation to starvation, with overlapping regulation of cell adhesion, membrane dynamics, RNA processing, and metabolic homeostasis.

### 3.8. Identification of Overlapping Genes Between PNR-Related Genes and DEU-Associated Genes

**Figure 5:**
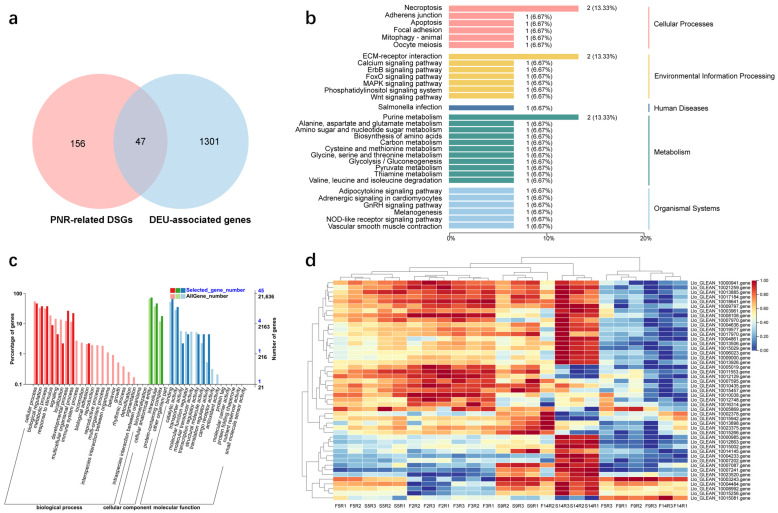
(**a**) Venn diagram showing that the overlap between PNR DSGs and PNR DEU-associated genes yielded 47 shared genes. (**b**) KEGG enrichment analysis of the 47 overlapping genes. (**c**) GO enrichment analysis of the 47 overlapping genes. (**d**) Hierarchical clustering heatmap of the normalized expression levels of the 47 overlapping genes across starvation and feeding samples. Red and blue indicate relatively high and low expression levels, respectively.

To determine the candidate genes associated with the PNR stage under starvation, the 203 genes identified from the S14 vs. S9 comparison were intersected with the 1348 DEU-associated genes identified from the same comparison. The analysis revealed 47 overlapping genes (Figure 5a), which retained genes supported by both gene-level splicing changes and exon-level evidence, thereby reducing false positives arising from any single analytical layer. This highlights a subset of robust late-starvation splicing candidates that warrant further experimental validation. The full list of these 47 genes is provided in Table A2.

Altered exon usage in the 47 overlapping PNR-related DSGs and DEU-associated genes may lead to diverse functional consequences, including protein domain loss, frameshift mutations, premature stop codons, and nonsense-mediated decay (NMD). Most alternatively spliced exons mapped to functional protein domains, implying that exon skipping or inclusion could modify protein structure and physiological function. These structural changes are likely to reshape the activity of stress sensing, cell adhesion and immune-related proteins and ultimately mediate larval responses to starvation at the PNR.

The results of the GO enrichment analysis indicated that the overlapping genes were involved mainly in metabolic processes, cellular processes, biological regulation, response to stimulus, signaling, developmental processes, and immune system processes, with enrichment in intracellular components and protein-containing complexes, as well as binding and catalytic activity (Figure 5c). The results of the KEGG analysis revealed enrichment in pathways related to ECM–receptor interactions, adherens junctions, phosphatidylinositol signaling, ErbB signaling, adipocytokine signaling, mitophagy, necroptosis, and several metabolic pathways (Figure 5b). A hierarchical clustering heatmap of normalized expression profiles was generated and revealed a distinct stage-specific expression pattern of these genes during the progression of starvation, with most genes showing markedly upregulated expression at the S14 stage, indicating dynamic regulation during the PNR (Figure 5d).

Among the identified genes, the DEU patterns of several genes, including *lama2*, *mbpa*, *zak*, and *nhsl2*, are analogous to those of AS, Furthermore, these genes exhibit high difference in PSI (|ΔPSI|) with statistically significant *p* values, indicating robust differential splicing between conditions. Ribotoxic stress is specifically detected in the *zak* gene. Under starvation conditions, amino acid deficiency and the accumulation of reactive oxygen species (ROS) may induce ribosomal damage, thereby activating *zak*. This activation can trigger the ribotoxic stress response (RSR) and subsequently engage the conserved p38/JNK MAPK signaling cascade, influencing cell fate decisions ranging from adaptive stress responses to apoptosis [33,34]. The *lama2* gene may help adapt to starvation stress through its role in ECM–receptor interactions and focal adhesion pathways. In support of this speculation, some studies have shown that *lama2* deficiency disrupts the organization of the extracellular matrix, cytoskeletal integrity, and cellular homeostasis and is associated with increased oxidative stress and DNA damage [35]. These findings suggest that *lama2* may help maintain the stability of the basement membrane and promote cell survival under nutrient deprivation conditions. The AS events of *mbpa* and *nhsl2* were also highly pronounced. The *mbpa* gene, associated with the functions of the myelin sheath and plasma membrane, may undergo changes in splicing related to structural maintenance and neural adaptation under starvation conditions. In contrast, *nhsl2*, which is associated mainly with developmental and cellular processes, may be involved in the starvation-induced regulation of cell differentiation and development. These distinct splicing profiles suggest that both genes may participate in the response to starvation through different biological pathways.

### 3.9. RT-PCR Validation of Key DSEs

**Figure 6:**
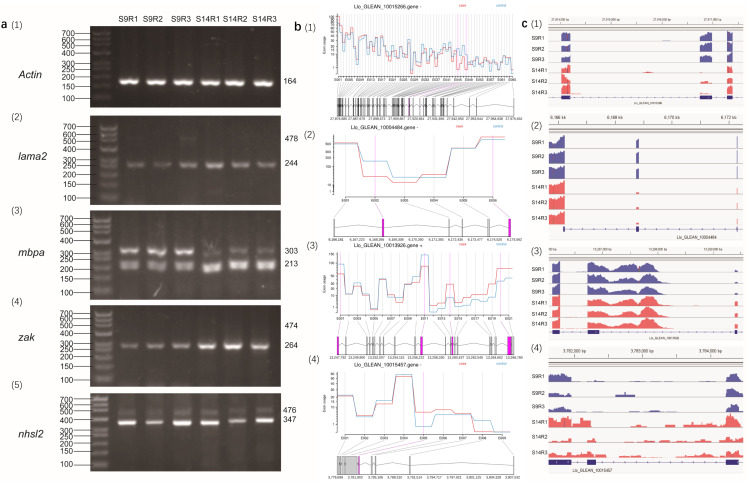
Validation of DSEs in candidate genes among the 47 overlapping genes in the comparison between S14 and S9 is presented. (**a**) Semi-quantitative RT-PCR analysis was performed to validate the AS isoforms of four candidate genes, (2) *lama2*, (3) *mbpa*, (4) *zak*, and (5) *nhsl2*, with (1) *Actin* serving as the internal control. For each gene, the DNA size marker is shown on the left. Lanes 1–3 represent three biological replicates from the S9 stage, and lanes 4–6 represent three biological replicates from the S14 stage (PNR). The expected sizes of the amplified products corresponding to specific splice isoforms are shown on the right. (**b**) The DEU profiles of the four genes based on the RNA-seq data are presented. where panels (1) to (4) correspond to *lama2*, *mbpa*, *zak*, and *nhsl2*, respectively. Red and blue lines indicate the S14 and S9 stages, respectively. In the gene models below each plot, the pink vertical bars indicate exons whose usage significantly differed between the two stages. (**c**) Integrative Genomics Viewer (IGV) snapshots showing RNA-seq read coverage across the alternatively spliced regions of the four genes, with panels (1) to (4) corresponding to *lama2*, *mbpa*, *zak*, and *nhsl2*, respectively. The blue tracks represent the S9 biological replicates (S9R1–S9R3), and the red tracks represent the S14 biological replicates (S14R1–S14R3). The differences in read depth and junction-spanning reads support the exon inclusion/skipping events detected by RT-PCR and DEU analysis.

Among the 47 overlapping genes obtained by intersecting the PNR-related DSGs with the DEU-associated genes in the S14 vs. S9 comparison, four genes, namely, *lama2*, *mbpa*, *zak*, and *nhsl2*, were selected for validation via RT-PCR analysis, with Actin serving as the internal control. The AS events validated in these genes were all SE events, which is consistent with the RNA-seq prediction results. Specifically, *lama2* (Chr8, exon 27,916,751–27,916,985, IncLevelDifference = 0.693), *mbpa* (Chr20, exon 6,168,747–6,168,837, IncLevelDifference = 0.538), and *zak* (Chr9, exon 13,256,734–13,256,944, IncLevelDifference = 0.334) showed greater exon inclusion in S14 than in S9, and *nhsl2* (Chr7, exon 3,782,193–3,782,322, IncLevelDifference = −0.435) presented lower exon inclusion (Table 3), incLevelDifference represents the difference in PSI between groups. The RT-PCR banding patterns matched the splice isoforms predicted from the RNA–seq data, and the differential splicing patterns among groups showed the same overall trends as those detected by transcriptome sequencing (Figure 6). These findings confirmed the reliability of the RNA-seq data and the robustness of the differential AS analysis.

### 3.10. Integrative Model of AS Dynamics During Nutritional Transition and Starvation-Induced PNR

**Figure 7:**
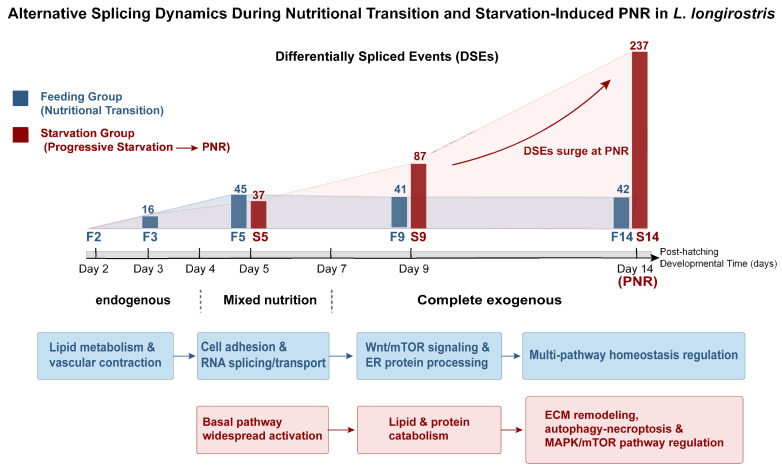
Integrative model of alternative splicing dynamics during nutritional transition and starvation-induced PNR in *L. longirostris* larvae.

To synthesize the stage-specific patterns described above, we constructed an integrative model summarizing the distinct trajectories of AS dynamics during successful nutritional transition and progressive starvation (Figure 7). In the feeding group, DSEs accumulated gradually across developmental stages, with the associated biological processes shifting sequentially from lipid metabolism and vascular contraction during endogenous nutrition (F2–F3), to cell adhesion and RNA splicing/transport during mixed nutrition (F5), then to Wnt/mTOR signaling and endoplasmic reticulum protein processing during complete exogenous feeding (F9), and finally to multi-pathway homeostatic regulation at the stabilization stage (F14). In contrast, the starvation group exhibited a markedly different pattern: DSEs remained relatively low during early starvation (S5) but increased sharply at the PNR stage (S14), with AS-mediated processes progressing from broad activation of basal pathways at S5, to lipid and protein catabolism at S9, and ultimately to extensive ECM remodeling, autophagy-necroptosis, and dysregulation of the MAPK/mTOR pathways at S14. This model suggests that alternative splicing dynamics are closely associated with nutritional transition and starvation-induced PNR in *L. longirostris* larvae.

## 4. Discussion

This study systematically characterized the dynamic regulatory network of AS during the transition from endogenous to exogenous nutrition in *L. longirostris* larvae. A total of 84,172 AS events were identified, among which SE events accounted for 93.4%. This splicing pattern is consistent with observations in zebrafish during adaptation to low temperatures [36], where SE is also the predominant type of AS, and in euryhaline fish species such as turbot during salinity adaptation [37], indicating that SE may represent a conserved and flexible regulatory strategy in fish.

During the feeding group comparisons, the number of DSEs increased progressively across developmental stages, indicating that splicing regulation became more active as the larvae advanced through ontogeny. Before the first feeding event, AS was associated mainly with processes related to lipid mobilization and cardiovascular maturation, suggesting that the larvae still relied heavily on maternal reserves and internal developmental programming [23]. As development proceeded into the mixed-nutrition stage, DSEs were increasingly enriched in pathways such as adherens junctions and Notch signaling, suggesting that splicing contributes to intestinal barrier formation and tissue differentiation [26]. At the stage of complete dependence on exogenous nutrition (Day 9), regulation of the mTOR and Wnt signaling pathways by AS marked the transition from morphogenesis to nutrition-dependent growth [28,29]. At the stable stage (Day 14), enrichment in aminoacyl-tRNA biosynthesis and tight junction pathways reflected that physiological homeostasis was achieved. Together, these results suggest that AS is not a secondary byproduct of transcriptional change but rather an active regulatory mechanism that coordinates developmental progression in *L. longirostris* larvae.

In the starvation group, DSEs increased sharply, with the highest degree of splicing activity observed at the PNR stage. This pattern suggests that starvation triggers a rapid and coordinated AS response to nutritional stress. The abrupt increase in DSEs may reflect the need to reprogram transcripts involved in cell adhesion, energy sensing, and metabolic remodeling to preserve cellular integrity and optimize resource allocation under nutrient limitation. The enrichment of stress-related pathways at the PNR further supports the idea that AS acts as an early molecular response to starvation-induced physiological imbalance. Cross-stage comparisons between starved and fed larvae further revealed that AS-associated remodeling progressed from membrane disorganization to stress adaptation and then to metabolic reprogramming, suggesting that AS functions as a dynamic regulatory layer that coordinates developmental maturation and starvation survival.

Among the PNR-related DSGs associated with DEU-associated genes, most were significantly upregulated at the S14 stage, indicating that the onset of PNR coincides with a marked reorganization of the splicing network. SE events in *zak* may enhance its function as a ribotoxic stress sensor, potentially activating the ribotoxic stress response and downstream p38/JNK signaling under conditions of amino acid deprivation and oxidative stress [33,34]. In parallel, changes in the splicing of *lama2* may weaken basement membrane stability and affect tissue integrity, thereby accelerating structural deterioration during prolonged starvation. These results suggest that the PNR is accompanied by coordinated splicing changes in genes related to stress sensing and tissue maintenance, which may contribute to the decline in larval survival capacity [35]. The reliability of these splicing events was confirmed by RT-PCR analysis, which may provide candidate markers for future studies.

With respect to species specificity, *L. longirostris* reached its PNR on Day 14 post-hatching. This latent period varies considerably among teleosts: rapidly developing species such as silver perch (*Leiopotherapon plumbeus*) reach PNR within 48–96 h, whereas cold-water species, including the Chinese sturgeon (*Acipenser sinensis*) and walleye pollock (*Gadus chalcogrammus*), survive for at least 14 days [38,39,40].

These findings have direct practical implications for aquaculture. First, the PSI values of PNR marker genes, such as *lama2* and *zak*, may provide candidate markers for future studies. Second, the AS-regulated pathways identified here, especially mTOR, insulin signaling, and adhesion-related pathways, may provide targets for improving stage-specific feeding strategies. In particular, enhancing intestinal maturation during the mixed-nutrition stage may improve first-feeding success and reduce mortality. However, this study has several limitations. First, the roles of the identified isoforms were inferred mainly from transcriptomic and splicing evidence, and direct mechanistic validation remains limited. We also lack isoform expression analysis, isoform-specific functional annotation, and independent physiological verification for *zak*, *lama2*, *mbpa*, and *nhsl2*. Second, the absence of complementary physiological metrics (e.g., digestive enzymes and oxidative stress) and environmental covariates (e.g., temperature and dissolved oxygen) limits our biological interpretation and may confound nutritional stress responses. Therefore, additional experimental studies are needed to confirm the causal roles of these AS events in larval development and starvation adaptation. Researchers should focus on the following crucial directions: (i) conducting exon knockout experiments based on the CRISPR technique to determine the functional disparities among the splice isoforms of *zak* and *lama2*; (ii) exploring isoform expression and functions of core candidate genes and perform relevant physiological validation; and (iii) examining the interactive effects of environmental factors and detailed physiological profiling on the regulation of AS, thereby establishing a multidimensional framework for assessing larval quality.

## 5. Conclusions

Alternative splicing (AS) is pervasive and stage-specific; 84,172 AS events were identified in this study, with SE being the predominant type of AS. During normal development, AS sequentially regulates the mobilization of yolk lipids, formation of the intestinal barrier, mTOR/Wnt-mediated growth signaling, and physiological homeostasis. In this study, starvation induced progressive reprogramming of the splicing process, as the number of DSEs increased progressively along the trajectory from amino acid mobilization and mitophagy through lipid/protein catabolism and necroptosis to disruption of cell adhesion and global metabolic dysregulation. Most of the 47 PNR-related DSGs were upregulated at the S14 stage. SE events in *zak* may activate the conserved RSR-p38/JNK cascade. Along with *lama2*-mediated destabilization, these events may accelerate tissue degradation and serve as early PNR markers.

To summarize, AS serves as a unified post-transcriptional control layer that orchestrates developmental nutritional transition and mediates the molecular pathophysiology of starvation-induced reduction in the population of *L. longirostris* larvae. These findings provide novel insights into larval survival strategies and may provide candidate markers for future studies investigating splicing-based biomarkers and regulatory mechanisms relevant to larval development and hatchery management.

## Figures and Tables

**Table 1:** Sequences of the primers used for RT-PCR validation in *L. longirostris.*

Primer Name	Annealing Temperature (°C)	bp	Sequence (5′ to 3′)
lama2-F	60	22	TACGGAGGACCAAACTGTGAAA
lama2-R	60	21	CGAACGACTTAGAGCCGAATG
mbpa-F	60	24	TGGAGGTGATAAGAAGAAAAGAGG
mbpa-R	60	20	CGTTTGGGTGGAGAACGAGA
zak-F	60	22	CAACTCCTTCCTGCAAAACAAA
zak-R	60	22	ATTTCGCACTCTACACCGTCTG
nhsl2-F	60	21	GGGTAACTGGGCAGACATTCC
nhsl2-R	60	24	ACAGGCACCTTGTATCAGATCAAC
Actin-F	60	20	GCTACAGCTTCACTACCACA
Actin-R	60	20	GCCAATGGTGATGACCTGTC

**Table 2:** The number of DSEs and DSGs detected in each comparison.

Comparison Group	SE	MXE	Total DSEs	DSGs
F3 vs. F2	13	3	16	14
F5 vs. F3	32	13	45	33
F9 vs. F5	35	6	41	39
F14 vs. F9	35	7	42	40
S5 vs. F3	30	7	37	33
S9 vs. S5	74	13	87	77
S14 vs. S9	202	35	237	203
S5 vs. F5	13	11	24	24
S9 vs. F9	52	13	65	54
S14 vs. F14	192	34	226	189

**Table 3:** Detailed information on the chromosomal location, strand, exon coordinates, *p* value, FDR, inclusion level difference, and event type of the four randomly selected genes (*lama2*, *mbpa*, *zak*, and *nhsl2*) among the 47 overlapping genes in the comparison between S14 and S9 is presented.

GeneID	Chr	Strand	exonStart_0base	exonEnd	*p* Value	FDR	IncLevelDifference	Event_Type
*lama2*	8	−	27,916,751	27,916,985	0	0	0.693	SE
*mbpa*	20	−	6,168,747	6,168,837	0	0	0.538	SE
*zak*	9	+	13,256,734	13,256,944	4.90 × 10^−12^	5.93 × 10^−10^	0.334	SE
*nhsl2*	7	−	3,782,193	3,782,322	0.000159928	0.002514031	−0.435	SE

## Data Availability

The raw transcriptomic sequencing data generated in this study have been deposited in the National Center for Biotechnology Information (NCBI) Sequence Read Archive (SRA) database under the BioProject accession number PRJNA1445138.

## Abbreviations

The following abbreviations are used in this manuscript:

AS	Alternative splicing
PNR	Point of no return
rMATS	replicate multivariate analysis of transcript splicing
DSEs	Differentially spliced events
DSGs	Differentially spliced genes
SE	Skipped exons
GO	Gene Ontology
KEGG	Kyoto Encyclopedia of Genes and Genomes
QC	Quality control
NCBI	National Center for Biotechnology Information
SRA	Sequence Read Archive
FPKM	Fragments Per Kilobase of transcript per Million mapped fragments
A5SS	Alternative 5′ splice site
A3SS	Alternative 3′ splice site
RI	Retained intron
MXE	Mutually exclusive exons
PSI	Percent spliced-in
FDR	False discovery rate
DEU	Differential exon usage
BP	Biological process
CC	Cellular component
MF	Molecular function
mTOR	Mammalian target of rapamycin
MAPK	Mitogen-activated protein kinase

## Appendix A

**Table A1:** Summary of RNA sequencing quality control metrics for all samples.

Samples	Clean Reads	Clean Bases	GC Content	% ≥ Q30
F14R1	21,839,593	6,551,877,900	50.07%	97.04%
F14R2	23,887,406	7,166,221,800	49.19%	97.13%
F14R3	42,258,003	12,677,400,900	50.09%	97.45%
F2R1	21,717,508	6,515,252,400	48.37%	96.99%
F2R2	21,699,276	6,509,782,800	48.25%	97.08%
F2R3	22,185,752	6,655,725,600	48.54%	96.99%
F3R1	20,259,523	6,077,856,900	48.37%	97.13%
F3R2	24,717,353	7,415,205,900	48.54%	97.66%
F3R3	20,361,789	6,108,536,700	47.89%	97.23%
F5R1	51,359,866	15,407,959,800	48.90%	97.43%
F5R2	26,968,540	8,090,562,000	48.57%	97.32%
F5R3	30,219,038	9,065,711,400	49.26%	97.27%
F9R1	22,550,344	6,765,103,200	48.69%	97.27%
F9R2	21,997,766	6,599,329,800	48.94%	97.13%
F9R3	22,055,964	6,616,789,200	49.53%	97.13%
S14R1	23,513,078	7,053,923,400	47.77%	96.97%
S14R2	21,817,375	6,545,212,500	47.60%	96.87%
S14R3	54,050,253	16,215,075,900	47.00%	97.26%
S5R1	24,228,278	7,268,483,400	48.20%	97.21%
S5R2	21,906,075	6,571,822,500	48.47%	97.18%
S5R3	21,931,357	6,579,407,100	48.70%	97.18%
S9R1	22,040,343	6,612,102,900	48.58%	97.04%
S9R2	22,081,556	6,624,466,800	48.69%	97.00%
S9R3	22,031,558	6,609,467,400	48.70%	97.03%

**Table A2:** 47 PNR-core genes identified from DSG and DEU integration.

Gene_ID
Llo_GLEAN_10010038.gene
Llo_GLEAN_10009797.gene
Llo_GLEAN_10012663.gene
Llo_GLEAN_10000941.gene
Llo_GLEAN_10019577.gene
Llo_GLEAN_10018641.gene
Llo_GLEAN_10007202.gene
Llo_GLEAN_10015029.gene
Llo_GLEAN_10004636.gene
Llo_GLEAN_10017184.gene
Llo_GLEAN_10004484.gene
Llo_GLEAN_10013506.gene
Llo_GLEAN_10023520.gene
Llo_GLEAN_10015457.gene
Llo_GLEAN_10015256.gene
Llo_GLEAN_10004233.gene
Llo_GLEAN_10010435.gene
Llo_GLEAN_10004861.gene
Llo_GLEAN_10005869.gene
Llo_GLEAN_10017970.gene
Llo_GLEAN_10012748.gene
Llo_GLEAN_10015002.gene
Llo_GLEAN_10011553.gene
Llo_GLEAN_10007087.gene
Llo_GLEAN_10003961.gene
Llo_GLEAN_10013926.gene
Llo_GLEAN_10015266.gene
Llo_GLEAN_10005019.gene
Llo_GLEAN_10012129.gene
Llo_GLEAN_10000985.gene
Llo_GLEAN_10013885.gene
Llo_GLEAN_10002378.gene
Llo_GLEAN_10008108.gene
Llo_GLEAN_10023375.gene
Llo_GLEAN_10007595.gene
Llo_GLEAN_10003243.gene
Llo_GLEAN_10006023.gene
Llo_GLEAN_10007970.gene
Llo_GLEAN_10016314.gene
Llo_GLEAN_10015942.gene
Llo_GLEAN_10009000.gene
Llo_GLEAN_10008992.gene
Llo_GLEAN_10014145.gene
Llo_GLEAN_10007241.gene
Llo_GLEAN_10013898.gene
Llo_GLEAN_10015081.gene
Llo_GLEAN_10021259.gene
